# Dissolution of β-C_2_S Cement Clinker: Part 2 Atomistic Kinetic Monte Carlo (KMC) Upscaling Approach

**DOI:** 10.3390/ma15196716

**Published:** 2022-09-27

**Authors:** Mohammadreza Izadifar, Neven Ukrainczyk, Khondakar Mohammad Salah Uddin, Bernhard Middendorf, Eduardus Koenders

**Affiliations:** 1 Institute of Construction and Building Materials, Technical University of Darmstadt, Franziska-Braun-Str. 3, 64287 Darmstadt, Germany; 2 Department of Structural Materials and Construction Chemistry, University of Kassel, Mönchebergstraße 7, 34125 Kassel, Germany

**Keywords:** cement dissolution, belite cement clinker, atomistic kinetic Monte Carlo, upscaling approach, dissolution rate, crystal defects, periodic boundary conditions (PBC)

## Abstract

Cement clinkers containing mainly belite (β-C_2_S as a model crystal), replacing alite, offer a promising solution for the development of environmentally friendly solutions to reduce the high level of CO_2_ emissions in the production of Portland cement. However, the much lower reactivity of belite compared to alite limits the widespread use of belite cements. Therefore, this work presents a fundamental atomistic computational approach for comprehending and quantifying the mesoscopic forward dissolution rate of β-C_2_S, applied to two reactive crystal facets of (100) and ($\bar{1}00$). For this, an atomistic kinetic Monte Carlo (KMC) upscaling approach for cement clinker was developed. It was based on the calculated activation energies (ΔG*) under far-from-equilibrium conditions obtained by a molecular dynamic simulation using the combined approach of ReaxFF and metadynamics, as described in the Part 1 paper in this Special Issue. Thus, the individual atomistic dissolution rates were used as input parameters for implementing the KMC upscaling approach coded in MATLAB to study the dissolution time and morphology changes at the mesoscopic scale. Four different cases and 21 event scenarios were considered for the dissolution of calcium atoms (*Ca*) and silicate monomers. For this purpose, the (100) and ($\bar{1}00$) facets of a β-C_2_S crystal were considered using periodic boundary conditions (PBCs). In order to demonstrate the statistical nature of the KMC approach, 40 numerical realizations were presented. The major findings showed a striking layer-by-layer dissolution mechanism in the case of an ideal crystal, where the total dissolution rate was limited by the much slower dissolution of the silicate monomer compared to *Ca*. The introduction of crystal defects, namely cutting the edges at two crystal boundaries, increased the overall average dissolution rate by a factor of 519.

## 1. Introduction

Concrete is the second most consumed material on Earth after water [1], and it plays a vital role in the construction industry. This is due to its excellent combination of strength, affordability, and shape moldability [2]. Driven by the construction industry’s enormous demand for the production of Portland cement (PC), it is responsible for roughly 5% of global anthropogenic CO_2_ emissions [3,4]. Thus, the production of cement clinker with a lower CO_2_ footprint and reduced energy consumption has become the major challenge for developing environmentally friendly concrete. The production of cement rich in β-C_2_S clinker is a beneficial solution due to its lower limestone demand, associated CO_2_ emissions, energy demand, and temperature increase at early-age hydration [5]. However, after alite (C_3_S) [6,7], β-C_2_S is considered the second most significant clinker phase (typically representing about 15%), and its lower reactivity has remained a major issue. Moreover, cements with a higher belite content have a slower rate of strength development. For instance, the compressive strength of concrete made from pure alite clinker (58.4 MPa) is 79% higher than a pure belite clinker (32.5 MPa) [8]. Therefore, it is first necessary to examine the reactivity of β-C_2_S for the five most distinguished reactive crystal facets [4,9,10,11].

Atomistic simulations using a molecular dynamics (MD) method, boosted by the application of reactive force fields (ReaxFF) and metadynamics (MetaD), offer a powerful way of investigating how the atomistic processes involved in the dissolution reaction transition states of crystal sites affect the dissolution kinetics. However, they are strongly limited in simulating processes for individual crystal sites. As an upscaling approach, kinetic Monte Carlo (KMC) simulations are promising for mineral phase dissolution over much larger timescales and with mesoscopic crystal sizes. In this study, we developed an atomistic KMC upscaling approach for cement clinker dissolution. The major idea of the KMC computational upscaling method is the computation of the mesoscopic forward dissolution rate (*R*, s^−1^) under far-from-equilibrium conditions based on the activation energies obtained by molecular dynamics (MD) [12,13] from the Part 1 β-C_2_S (belite) case study [14]. In fact, many studies have been carried out to investigate the dissolution/precipitation of minerals by a KMC upscaling approach. Most recently, Izadifar et al. [15] investigated the mesoscopic forward dissolution rate of portlandite for the hexagonal crystals comprised of four different facets (100 or $\bar{1}00$, 010 or $0\bar{1}0$, $\bar{1}10$ or 1$\bar{1}0$, and 001 or 00$\bar{1}$), resulting in a total of 16 different atomistic event scenarios under far-from-equilibrium conditions based on the atomistic activation energies (ΔG*) computed by the MetaD molecular dynamic (MD) computational approach (Part 1) [16]. Results for portlandite showed that during the dissolution of almost two thousand calcium atoms (*Ca*) crystal sites, contributions from the 100 or $\bar{1}00$, 010 or $0\bar{1}0$, and 001 or 00$\bar{1}$ facets were hardly observed. In contrast, 96% of dissolutions took place on the 010 or $0\bar{1}0$ facets, and only a small contribution of 4% was due to dissolution on the $\bar{1}10$ or 1$\bar{1}0$ facets. The mesoscopic forward dissolution rate for the reactive facets of 010 or $0\bar{1}0$ was reported to be 1.0443 mol/(s cm^2^). The atomistic entropy contributions (ΔS*) have also been reported according to the differences between the enthalpy (Δ*Ha*) (which was computed at 0 K by the nudged elastic band (NEB) method [17,18,19,20] within a DFT [21] computational approach, as defined in the Vienna ab initio simulation package (VASP) [22,23,24,25,26,27]) and the total atomistic activation energy (ΔG*) (obtained by the MetaD computational approach at 298 K). Martin et al. [28] investigated the concentration dependence of the dissolution rate in the KMC model for the dissolution of a simple Kossel crystal, describing the experimentally observed sigmoid dependence of the dissolution rate against the concentration, i.e., the macroscopic free-energy driving force (Δ*G*). Moreover, Martin et al. [29] recently reported the dissolution of a quartz crystal using the atomistic KMC model based on the joint reproduction of the bond-by-bond reaction rates *R_dis_* and *E_a_*. However, to upscale the dissolution rates for complex crystal structures such as belite, far-from-equilibrium conditions have to be considered first, thus neglecting any solution concentration (backward) effects. The upscaling result thus computes the forward dissolution rate constant.

The kinetics of crystal dissolution (or growth) can be surface- or diffusion-controlled, while in a steady state situation the rate of mass transport from the crystal surface into the (bulk) solution must inevitably correspond to the reaction rate at the surface [30]. Here, the focus is only on the surface reaction. The kinetics of crystal dissolution/growth are controlled by the atomistic processes at the surface of the crystals. In many of these scenarios, crystal dissolution follows an ordered mechanism, the theory being based on the step retreat (or advance in the case of growth) at the surface. Under equilibrium conditions, adatoms (individual sites adsorbed on the crystal surface) and terraces dissolve to form a flat surface [28]. With an increased degree of undersaturation, the dissolution nuclei become (a) vacancies (single empty sites) on the crystal surface and (b) dislocations (holes/tunnels into the crystal, with an opening size of a single crystal site). This can lead to two distinct dissolution mechanisms: (1) the opening of pits and/or (2) step retraction [28]. In order to computationally reveal the driving mechanisms, atomistic simulations should be upscaled to simulate mesoscopic dissolution rates. Thus, the main objective of the present study was to develop a primary physical/chemical bridging model for the initial mesoscopic forward dissolution rate computation of β-C_2_S using the KMC upscaling approach under far-from-equilibrium conditions. The modeling approach was demonstrated to simulate the dissolution of two reactive facets of (100) and ($\bar{1}00$). To observe the effect of crystal defects on the dissolution rates, a comparison was drawn with the ideal crystal morphology, wherein periodic boundary conditions (PBCs) were applied along the Y and Z axes and PBCs were removed along the Y axis. This represented cutting the crystal perpendicular to the Y axis, with two sides having plane defects. To implement the KMC upscaling approach, the atomistic forward dissolution rates ($r_{D}$) for all the most important event scenarios needed to be computed. These inputs were provided as results from the Part 1 β-C_2_S case study employing the MetaD–ReaxFF MD computational approach. The results of the MD simulations were in the form of activation energies, obtained for the dissolution of *Ca* and silicate monomer. Moreover, they were computed for four different cases, depending on the possible neighboring scenarios. Having *Ea* as the input, the atomistic forward reaction rates were calculated based on the transition-state theory (TST) [30]. Thus, to implement the KMC upscaling approach for the mesoscopic total forward dissolution rate, we needed to compute the individual atomistic forward reaction rates ($r_{D}$) for different event scenarios. These were obtained using an Arrhenius-like equation based on the TST (Equation (1)), applying the activation energies (ΔG*) obtained from the MetaD–ReaxFF MD calculations:

(1) $$\;r_{D}=\frac{k_{B}T}{h}\;exp^{-\frac{\Delta G^{*}}{RT}}$$

where $k_{B}$ is the Boltzmann constant, *h* is the Plank’s constant, ΔG* is the free energy of activation computed by the MetaD–ReaxFF molecular dynamic computational approach (inputs form the Part 1 paper), *R* is the gas constant, and *T* is the temperature. 

**Figure 1:**
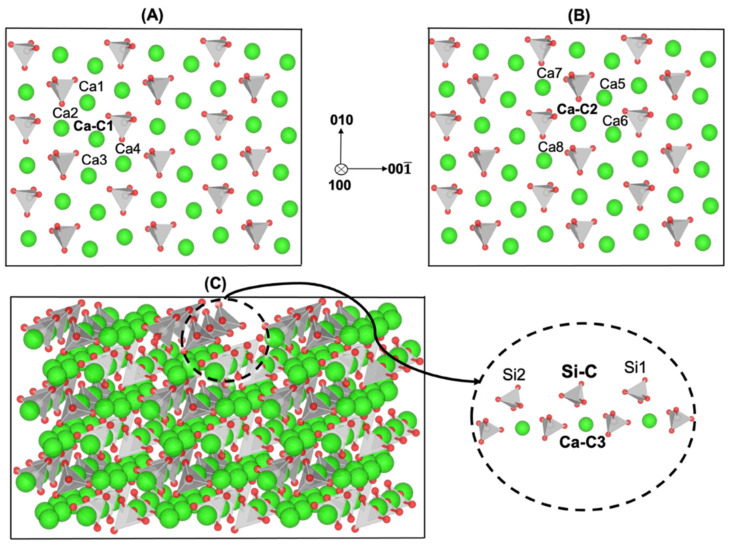
Illustration of four different cases for dissolution of Ca-C1, Ca-C2, Ca-C3, and Si-C depending on the existing nearest neighbors. (**A**,**B**) The dissolution of *Ca*, namely Ca-Ca1 and Ca-C2, from the row of *Ca* depending on the four existing neighbors related to cases 1 and 2, respectively. (**C**) The dissolution of Si-C (case 3) depending on the two silicate neighbors (Si1 and Si2), as well as Ca-C3 dissolution (case 4) depending on the existence of the upper Si atom (Si-C).

An example of the different atomistic dissolution events is shown in Figure 1, demonstrating how the different crystal sites (*Ca* or silicate monomer) can experience different scenarios depending on the neighbor arrangements, which change during the dissolution process (if neighbors are dissolved, their *Ea* changes, i.e., the atomistic rate of the central site). 

## 2. Methods and Computational Models

As described in this section, the dissolution mechanism and rate of cement clinker β-C_2_S were studied using an atomistic KMC upscaling approach on the (100) and ($\bar{1}00$) crystal facets. For this, the activation energies for the dissolution of *Ca* and silicate monomer were taken from the Part 1 paper (in the same Special Issue), considering a total of 21 different event scenarios depending on the existing neighbors. In this work, based on the transition-state theory (TST), the atomistic forward reaction rates ($r_{D}$) for different event scenarios were calculated (Equation (1)). Then, by implementing the atomistic kinetic Monte Carlo (KMC) upscaling approach in MATLAB, the dissolution time and morphology changes at the mesoscopic scale were thoroughly investigated. 

### 2.1. Atomistic Model Preparation for Computation of Reaction Rates 

To implement the KMC upscaling approach, the atomistic forward reaction rates ($r_{D}$) for various atomistic event scenarios had to be computed based on the free energy of activation. In this study, 4 different cases were considered depending on the different atomistic event scenarios. Figure 1C shows the monoclinic crystal structure of β-C_2_S. As shown in Figure 1A (1st case), the dissolution of Ca-C1 from the row of *Ca* (at the same level on the X axis) depended on the existence of 4 Ca neighbors (Ca1, Ca2, Ca3, and Ca4). Thus, 7 different atomistic event scenarios were considered based on the combination of the 4 remaining *Ca* neighbors. Figure 1B (2nd case) shows that the dissolution of Ca-C2 depended on the existence of 4 *Ca* neighbors (Ca5, Ca6, Ca7, and Ca8), resulting in the formation of 7 different event scenarios. After the dissolution of all 5 *Ca* neighbors for each central silicate tetrahedra (Si), by applying 3 consecutive silicate monomers on the same layer on the facets of (100) and ($\bar{1}00$), the third case concerning the dissolution of the central silicate monomer (Si-C) could be defined. As the silicate monomer had two neighbors, this resulted in three dissolution scenarios. Therefore, concerning the third case, three different event scenarios were created depending on the existing neighbors: the presence of both left and right neighbors (Si1 and Si2), the presence of either the left or right neighbor alone, and the absence of both left and right neighbors. The third and fourth cases are shown in Figure 1C. The fourth case concerned *Ca* dissolution (Ca-C3) from the lower layer, which was completely dependent on the third case. In other words, only when the conditions of the third case were met did the possibility of the dissolution of *Ca* (Ca-C3) open up, as the *Ca* was positioned directly below the silicate monomer (Si-C). Regarding the dissolution of Ca-C3 in the fourth case, 2 different event scenarios could be considered: the presence of Si-C and the absence of Si-C. The atomistic activation energies (ΔG*) of all the event scenarios involving *Ca* and silicate monomer dissolutions and their corresponding atomistic forward reactions rate ($r_{D}$) computations according to Equation (1) are shown in Table 1, Table 2, Table 3 and Table 4.

### 2.2. Atomistic Kinetic Monte Carlo Upscaling Approach: Implementation in MATLAB 

After computing the atomistic forward reaction rates ($r_{D}$) of *Ca* and silicate monomer dissolution on the facets of (100) and ($\bar{1}00$) for 21 different event scenarios, a MATLAB code [15] was developed for the implementation of the kinetic Monte Carlo (KMC) upscaling approach. For this, the mesoscopic scale was represented by a supercell consisting of 6048 atoms and 2724 (both *Ca* and silicate) sites. To implement the KMC algorithm, we initially needed to compute the total atomistic forward reaction rates $\left(k_{tot}\right)$ according to Equation (2) for all sites that were exposed to the aqueous media (i.e., the surface sites *p*), considering their event (neighboring) scenarios. It was essential to update all the surface sites after each event (i.e., the dissolution of each site) to distinguish the newly exposed surface sites.

(2) $$k_{tot}=\overset{N_{p}}{\underset{p=1}{\sum }}k_{p}\;$$

After this, we needed to normalize the rate of each event scenario to compute the probability of each event on a scale from 0 to 1, which was multiplied by the number of sites and then divided by $k_{tot}$. To determine the event probability, a random $\zeta_{1}$ value (between 0 to 1) had to be generated and, consequently, a random dissolution site was selected by choosing a random integer number (index) and applying it to the selected event. Finally, according to Equation (3), the time of dissolution for a selected site (Δt) was computed by the division of the second random $\zeta_{2}$ value (between 0 to 1) by $k_{tot}$ (the total atomistic forward reaction rate).

(3) $$\Delta t=-\ln\left(\zeta_{2}\right)/k_{tot}$$

The development of the MATLAB code based on the KMC algorithm was divided into four major sections. (1) In a pre-processor, the input structure was prepared and read, i.e., a crystal was created with a list of positions for all particles. A supercell crystal of β-C_2_S, which had been prepared earlier, was imported into the MATLAB code. Initially, to enable the easy tracking of the atoms during the dissolution process, the position of each site was indexed. Sites were separated into two groups: inner sites (the 010 or $0\bar{1}0$ and 001 or 00$\bar{1}$ facets were also distinguished as block sites) and outer sites, i.e., surface sites (the 100 and $\bar{1}00$ facets/sides available for dissolution). The sites were separated because of the dissolution process, which only occurred on the outer sites (the 100 and $\bar{1}00$ facets/sides) that were exposed to the surrounding aqueous media (pure water). (2) By employing an event processor, the execution of individual events was kept track of at the system level, and updates were implemented after changes in the system. Here, we needed to update the inner sites that had become part of the (100) and $\left(\bar{1}00\right)$ facets/sides, e.g., after the dissolution of neighbors from the outer sites (the (100) and $\left(\bar{1}00\right)$ facets/sides). It is also worth mentioning that the rate of dissolution for each site depended on the number of (missing) neighbors. Therefore, according to the crystal morphology of β-C_2_S, a total of 21 event scenarios for the dissolution of *Ca* and silicate monomers on the (100) and ($\bar{1}00$) facets/sides could be considered. (3) Aided by a solver, the actual rejection-free KMC algorithm could be implemented for the probabilistic-based selection of an event scenario to be executed. We searched for the outer sites most likely to be dissolved based on the 21 event scenarios of the computed atomistic forward reaction rates (*r_D_*) for the dissolution of *Ca* and silicate monomers on the (100) and ($\bar{1}00$) facets/sides, as shown in Table 1, Table 2, Table 3 and Table 4. (4) After the dissolution of one site, it was necessary to inform all neighbor sites about this missing dissolved site. This automatically changed the blocked sites into outer (exposed) sites, which contributed to the list of possible processes to be selected for dissolution in the next time step. (5) After post-processing, the data were prepared for exporting and plotting purposes. Finally, the third and fourth processes had to be iterated consecutively until reaching a user-defined number of (time) steps or until all sites were dissolved.

## 3. Results and Discussion

### 3.1. Dissolution Mechanism for β-C_2_S Crystal by Applying Periodic Boundary Conditions (PBCs) along the Y and Z Axes

**Figure 2:**
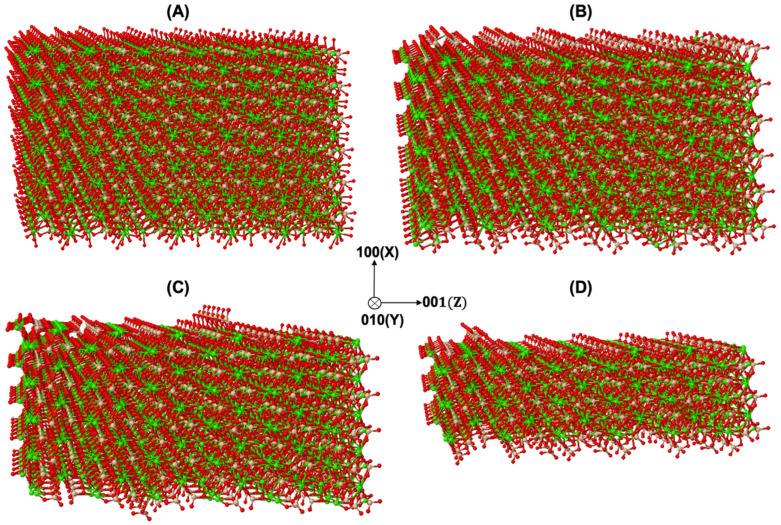
Evolution of β-C_2_S crystal during an atom-by-atom site dissolution KMC upscaling approach applied on the (100) and ($\bar{1}00$) facets. (**A**) Initial crystal morphology of β-C_2_S, consisting of 2724 sites. (**B**–**D**) Running of 272, 545, and 1362 dissolution steps resulted in dissolution of 10%, 20%, and 50% of the total number of crystal sites of the initial structure, respectively. Each step is representative of one site dissolution.

**Figure 3:**
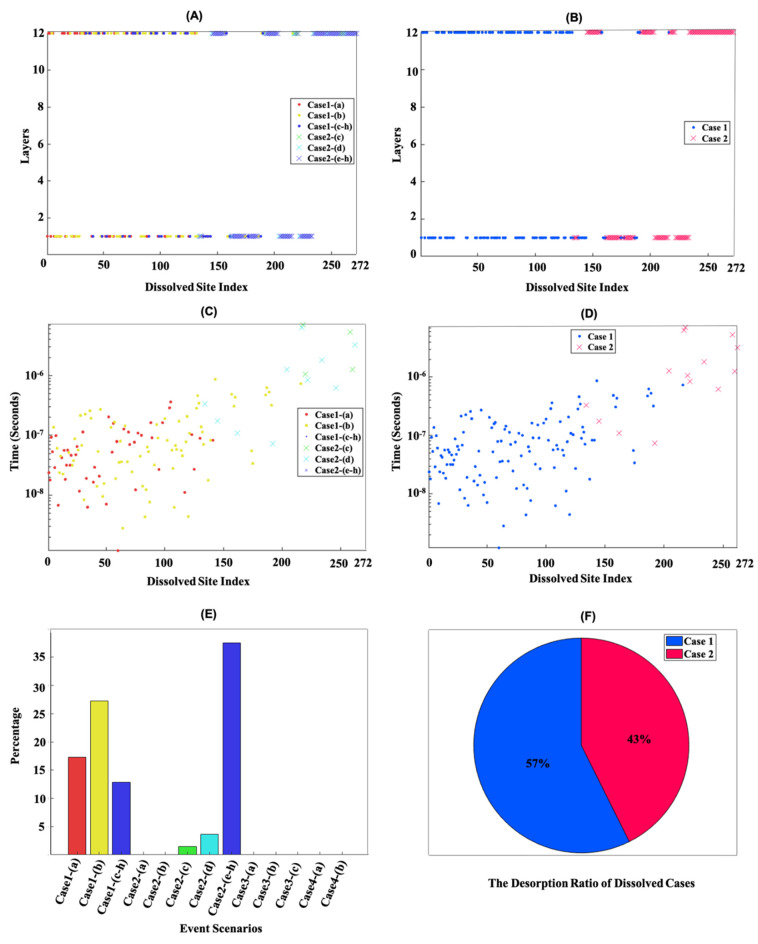
Pivotal details obtained through implementation of KMC upscaling approach after 10% β-C_2_S crystal dissolution (Figure 2B) by applying periodic boundary conditions (PBCs) along the Y and Z axes. (**A**,**B**) Illustration of dissolved *Ca* and silicate monomers for different event scenarios and cases applied to the 12 layers (along the X axis) as a function of the dissolved site index. (**C**,**D**) Representation of the dissolution time of each site for different event scenarios and cases. (**E**) The contribution of each event scenario as a percentage. (**F**) The contribution of each case after 10% dissolution of β-C_2_S crystal.

A snapshot of the morphology of the initial β-C_2_S crystal consisted of 2724 sites (Figure 2A). Figure 2B–D illustrate the site-by-site dissolution model of β-C_2_S by the temporal removal of *Ca* and silicate monomers on the (100) and $(\bar{1}00$) facets. Periodic boundary conditions (PBCs) were applied along the Y and Z axes. In each simulation step, one site was dissolved. By running the simulation for 272, 545, and 1362 dissolution steps, the simulation resulted in a crystal dissolution degree of 10%, 20%, and 50% (of the total number of crystal sites of the initial structure), respectively. The results showed that a layer-by-layer dissolution process had taken place due to the higher computed atomistic forward reaction rates of *Ca* (Table 1 and Table 2) and, consequently, the higher probability of *Ca* being selected during the implementation of the KMC upscaling approach. In order to examine the contribution of different cases and event scenarios during the dissolution of *Ca* and silicate monomers, more detailed information was extracted, e.g., after the dissolution of 272 sites, as shown in Figure 3. Figure 3A,B illustrate that the dissolution of *Ca* was initially accomplished on the 1st ($\bar{1}00$) and 12th (100) layers for all event scenarios from cases 1 and 2, except the event scenarios of (a) and (b) from case 2. As the PBCs were considered for the crystal morphology of β-C_2_S, and all sites had their neighbors, the initial 150 sites were mostly dissolved in event scenarios (a) and (b) related to case 1, due to the higher reaction rates and higher probability of dissolution computed for *Ca*. As time went on, with more site dissolutions, the probability of the involvement of event scenarios (c) and (d) from case 2 in the process of *Ca* dissolution increased. The selection of event scenario (d) from case 2 increased the probability of the selection of event scenario (c) from case 2 for *Ca* site dissolution in the same row and the same plane layer on the facets of (100) and $(\bar{1}00$), due to the higher atomistic rate in comparison to case 2 event scenario (d). In other words, the occurrence of event scenario (c) from case 2 depended on event scenario (d), requiring the absence of one *Ca* between silicate monomers. Finally, with the further dissolution of the *Ca* sites, other event scenarios with very fast reaction rates became involved in *Ca* dissolution (case1 (c–h) and case 2 (e–h)).

Figure 3C,D show the dissolution times of each individual site for different event scenarios and cases. These two figures prove our reported observations regarding the higher probability of *Ca* dissolution for event scenarios (a) and (b) from case 1, resulting in a shorter dissolution time of 10^−6^ to 10^−9^ in comparison to event scenarios (c) and (d) from case 2. It is also worth mentioning that the dissolution time of *Ca* sites in case 1 (c–h) and case 2 (e–h)) were not shown in Figure 3C,D due to their extremely high computed atomistic forward reaction rates, resulting in very rapid *Ca* dissolution.

Figure 3E shows the contribution of each event scenario as a percentage. As can be seen from case 2 in the bar graph, event scenario (a) in the presence of all neighbors and event scenario (b) in the absence of one *Ca* neighbor were not involved in the dissolution mechanism of *Ca*. The reason for this is that the dissolution of the *Ca* sites in the presence of all neighbors could only take place through event scenario (a) from case 1, which has a much faster atomistic reaction rate (i.e., higher event probability) (1.0 × 10^5^ s^−1^) in comparison with case 2 (9.97 × 10^−17^ s^−1^). Likewise, the dissolution of *Ca* sites in the absence of one *Ca* neighbor only took place through event scenario (b) from case 1, due to the faster atomistic reaction rate (2.5 × 10^5^ s^−1^) in comparison with case 2 (4.61 × 10^−15^ s^−1^). Moreover, as indicated in Figure 3E, the contributions of case 3 to silicate monomer dissolution and case 4 to *Ca* dissolution were not observed. In fact, Si monomers were likely to be selected during the dissolution process when the dissolution of all *Ca* from cases 1 and 2 was complete, due to their high computed reaction rates. However, after the first silicate monomer was dissolved, the lower *Ca* from case 4 had a higher chance of being dissolved. Therefore, the dissolution mechanism of β-C_2_S on the (100) and $(\bar{1}00$) facets was a layer-by-layer process, as shown in Figure 2. Thus, the opening of pits, another distinct dissolution mechanism proposed in the literature [28], was not observed in our simulations, which clearly demonstrated and explained the atomistic reasons behind the step retreat mechanism. After a sequential number of step retreats across the surface, the dissolution rate could be calculated as *rate* = *v h*/*L*, where *v* is the step velocity, *h* is the step height, and *L* is the mean distance between surface steps [30]. According to the theory of crystal dissolution and growth, researchers could use this as a cornerstone equation to quantitatively describe the movement of atomic steps on the crystal surface.

**Figure 4:**
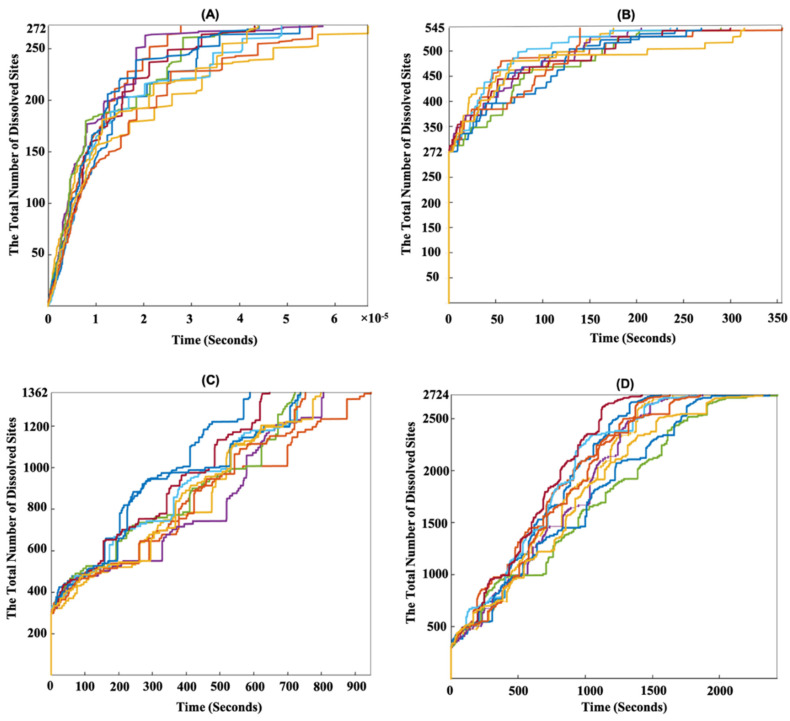
Time evolution of site dissolution for the β-C_2_S crystal by applying periodic boundary condition (PBCs) along the Y and Z axes for 10 numerical realizations (displayed with different colors) on the (100) and $(\bar{1}00$) facets. (**A**–**D**) After dissolution of 272, 545, 1362, and 2724 sites, the crystal had dissolved by 10%, 20%, 50%, and 100% of the total number of crystal sites of the initial structure, respectively.

Figure 3F shows that during the dissolution process, case 1 demonstrated a higher probability than case 2. This is because in case 1, higher atomistic reaction rates were computed for event scenarios (a–d) compared to those in case 2. Figure 4 shows the evolution over time of dissolution sites after the dissolution of 272, 545, 1362, and 2724 sites on the (100) and $(\bar{1}00$) facets. Periodic boundary conditions (PBCs) were applied along the Y and Z axes. In order to demonstrate the statistical nature of the KMC method, 10 numerical realizations were plotted. Figure 4A shows that the total time taken to dissolve 272 sites (10% dissolved) was between 2.5 × 10^−6^ and 6.5 × 10^−5^ s for the 10 numerical realizations. As shown in Figure 3C, the first 272 sites were related to the *Ca* dissolutions from cases 1 and 2. Therefore, due to the faster atomistic dissolutions of *Ca*, the overall dissolution rate at the mesoscopic scale increased. Figure 4B–D demonstrate that the silicate monomer with two neighbors (case 3 (a)) significantly slowed down the dissolution process due to its very slow atomistic rate of 1.5 × 10^−3^ s^−1^ (Table 3a), resulting in the dissolution of a single silicate monomer taking several seconds. However, the step-wise trend shown in Figure 4B–D was not only due to the very slow silicate monomer dissolution (case 3 (a)), but more importantly resulted from the sum of all (slow) surface sites (very low *k*_tot_ in Equation (3)), due to absence of rapid *Ca* sites on the surface.

It is also worth reporting that after the dissolution of one silicate monomer from a row, event scenario (b) from case 3 for the rest of the silicate monomers in the same row (i.e., with the absence of one silicate neighbor) had a higher probability of occurrence. This resulted in an incremental trend in the dissolution time of silicate monomers. The reason for this was the 5000-times larger atomistic reaction rate of a silicate monomer in the absence of one neighbor (case 3 (b)) in comparison to a silicate monomer in the presence of both neighbors (case 3 (a)).

### 3.2. Dissolution Mechanism for β-C_2_S Crystal by Applying Periodic Boundary Conditions (PBCs) along the Z Axis

**Figure 5:**
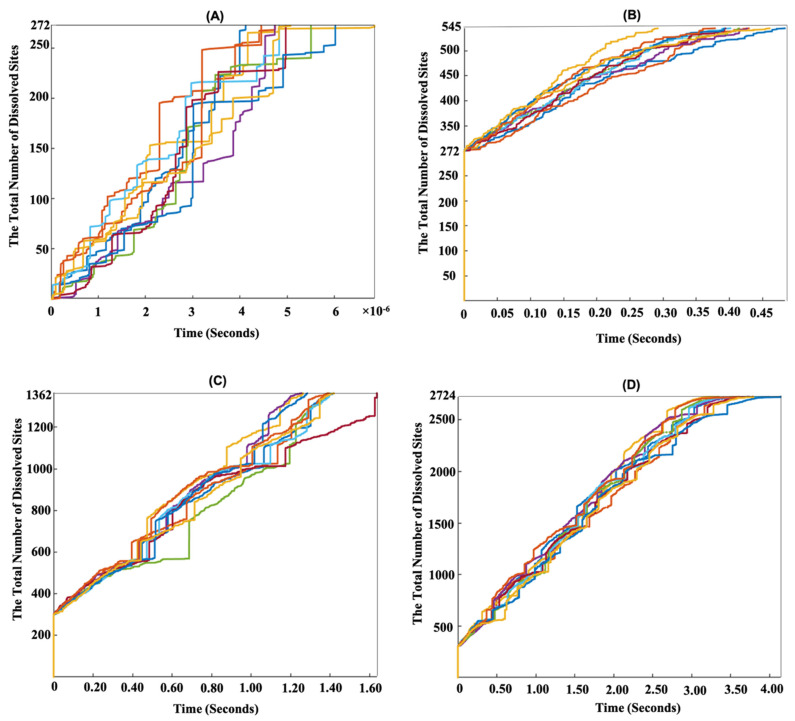
Time evolution of site dissolution for β-C_2_S crystal by applying periodic boundary conditions (PBCs) along the Z axis (defect border is applied along one axis) for 10 numerical realizations (displayed with different colors) on the (100) and $(\bar{1}00$) facets. (**A**–**D**) After dissolution of 272, 545, 1362, and 2724 sites representing 10%, 20%, 50%, and 100% of the total number of crystal sites of the initial structure, respectively.

To investigate the effect of crystal defects on dissolution rates, the PBCs (representing a semi-infinitive crystal) were simply replaced by cutting two edges (crystal boundaries), i.e., for two crystal facets (cutting the crystal perpendicular to the Y axis). First, the defect was investigated by applying the periodic boundary conditions (PBCs) only along the Z axis. Thus, the crystal was cut perpendicular to the Y axis, resulting in two cut crystal edges (boundaries). Figure 5 shows the time evolution of site dissolution (10 numerical realizations) after the dissolution of 272, 545, 1362, and 2724 sites. As expected, the contribution of the defect to the border increased the total dissolution time dramatically. To explain this in more detail, because PBCs were not applied along the Y axis, a defect on the borders in the direction of the Z axis increased the possibility for *Ca* sites to be dissolved rapidly at the beginning of the dissolution process due to the decreased number of *Ca* neighbors. This resulted in a 0.1 s reduction in the time taken (10^−6^ vs. 10^−5^ s) for the total dissolution of 272 sites (Figure 5A) in comparison to the system with PBCs for two borders in one direction (Figure 4A). To conclude, we compared the mesoscopic dissolution rates between the perfect and defect crystals. With the contribution of crystal defects on the borders along the Z axis, the total dissolution time of the whole β-C_2_S crystal was between 3 and 4.2 s for the 10 numerical realizations (Figure 5D). However, by considering a perfect (semi-infinitive) crystal using PBCs along the Z and Y axes, the total time taken to dissolve the whole β-C_2_S crystal was between 1400 and 2400 s for the 10 numerical realizations (Figure 4D). The contribution of the silicate monomers on the borders in the direction of the Z axis (with higher atomistic forward reaction rates due to missing one silicate monomer neighbor) should be regarded as the reason for the decremental trend in the total dissolution time for the crystal with a defect compared to the ideal crystal. In other words, the dissolution of an initial silicate monomer with both left and right neighbors in the ideal crystal (PBCs along the Y and Z axes) required more time due to the almost 5000-times lower atomistic forward reaction rate compared to the defective crystal with one missing silicate monomer neighbor (Table 3). In fact, when the PBCs were not applied along the Y axis, all silicate monomers on the borders in the direction of the Z axis could meet the conditions of case 3 event scenario (b) at the beginning of dissolution. As event scenario (a) from case 3, with its very low atomistic reaction rate, was no longer occurring, the total duration of site dissolution decreased.

To investigate the crystal size effects, a gigantic β-C_2_S crystal consisting of 48384 atoms and 21288 sites was fully dissolved. The behavior of the dissolution process did not change. 

## 4. Conclusions

Based on the results of this study, the following conclusions could be drawn. Initially, calcium atoms (*Ca*) was preferentially dissolved, with its higher probability of dissolution arising from its higher atomistic reaction rates compared to the silicates. Only after reaching *Ca* depletion in each layer did the dissolution of silicate monomers from the same layer take place. These results demonstrated that a layer-by-layer dissolution mechanism is responsible for the slow reactivity of belite clinker. 

The introduction of crystal defects by cutting the crystal perpendicular to the Y axis, i.e., resulting in two sides with plane defects caused by cutting the edges at two crystal boundaries, increased the overall average mesoscopic forward dissolution rate. Compared to the ideal crystal (semi-infinitive due to PBCs along the Y and Z axes), the cutting of the crystal sides resulted in a spectacular increase in the total mesoscopic forward dissolution rate; the total dissolution time required to fully dissolve the crystal decreased from 1400–2400 s (PBCs along the Y and Z axes) to 3–4.2 s (PBCs along the Z axis). The crystal defects reduced the total (mesoscale) dissolution time due to the (atomistic) contribution of the silicate monomer neighbors with an almost 5000-times higher atomistic forward reaction rate when the cut boundary was introduced (i.e., with one missing silicate monomer neighbor) compared to the ideal crystal.

The variability in the abovementioned dissolution times for both ideal and defective crystals resulted from the statistical nature of the KMC algorithm, captured by running 10 numerical realizations.

This paper paves the way for the upscaling of atomistic models to study the different dissolution behaviors of the individual facets of belite crystals. Although the obtained results are only a first step, in the long term they could lead to the production of more reactive belites in the manufacture of Portland cement clinker. This would allow the production of lower-energy-consumption and high-performance cements with a reduced CO_2_ footprint.

## Figures and Tables

**Table 1:** Atomistic activation energy (ΔG*) [14] and the calculated atomistic forward reaction rates ($r_{D}$) (using Equation (1)) for the Ca-C1 dissolution on the (100) and ($\bar{1}00$) facets for 8 different event scenarios applied in case 1. Ca-C1 dissolution (Figure 1A): (a) in the presence of Ca1, Ca2, Ca3, and Ca4; (b) in the presence of Ca2, Ca3, and Ca4; (c) in the presence of Ca2 and Ca4; (d) in the presence of Ca2 and Ca3; (e) in the presence of Ca1 and Ca3; (f) in the presence of Ca3; (g) in the presence of Ca2; and (h) in the absence of all 4 *Ca* neighbors.

Figure 1A	(a)	(b)	**(c)**	(d)	(e)	(f)	(g)	(h)
ΔG* (kJ/mol) [14]	44.61	42.20	0.00	0.00	0.00	0.00	0.00	0.00
*r_D_* (s^−1^)	1.0 × 10^5^	2.5 × 10^5^	$6.2\times 10^{12}$	$6.2\times 10^{12}$	$6.2\times 10^{12}$	$6.2\times 10^{12}$	$6.2\times 10^{12}$	$6.2\times 10^{12}$

**Table 2:** Atomistic activation energy (ΔG*) [14] and the calculated atomistic forward reaction rates ($r_{D}$(Equation (1)) for the dissolution of Ca-C2 on the (100) and ($\bar{1}00$) facets for 8 different event scenarios applied in case 2. Ca-C2 dissolution (Figure 1B): (a) in the presence of Ca5, Ca6, Ca7, and Ca8; (b) in the presence of Ca5, Ca7, and Ca8; (c) in the presence of Ca5, Ca6, and Ca7; (d) in the presence of Ca7, and Ca8; (e) in the presence of Ca5 and Ca6; (f) in the presence of Ca7; (g) in the presence of Ca6; and (h) in the absence of all 4 *Ca* neighbors.

Figure 1B	(a)	(b)	(c)	(d)	(e)	(f)	(g)	(h)
ΔG* (kJ/mol) [14]	164.30	154.80	44.06	52.06	0.00	0.00	0.00	0.00
*r_D_* (s^−1^)	9.97 × 10^−17^	4.61 × 10^−15^	$1.17\times 10^{5}$	$4.7\times 10^{3}$	$6.2\times 10^{12}$	$6.2\times 10^{12}$	$6.2\times 10^{12}$	$6.2\times 10^{12}$

**Table 3:** Atomistic activation energy (ΔG*) [14] and the calculated atomistic forward reaction rates ($r_{D}$) for the Si-C dissolution on the (100) and ($\bar{1}00$) facets for 3 different event scenarios applied in case 3. Si-C dissolution (Figure 1C): (a) in the presence of Si1 and Si2; (b) in the presence of Si1 or Si2; and (c) in the absence of both Si1 and Si2.

Figure 1C	(a)	(b)	(c)
ΔG* (kJ/mol) [14]	89.00	67.70	0.00
*r_D_* (s^−1^)	1.50 × 10^−3^	8.47	$6.20\times 10^{12}$

**Table 4:** Atomistic activation energy (ΔG*) [14] and the calculated atomistic forward reaction rates ($r_{D}$) (Equation (1)) for the Ca-C3 dissolution on the (100) and ($\bar{1}00$) facets for 2 different event scenarios applied in case 4. Ca-C3 dissolution (Figure 1C): (a) in the presence of Si-C and (b) in the absence of Si-C.

Figure 1C	(a)	(b)
ΔG* (kJ/mol) [14]	161.90	44.30
*r_D_* (s^−1^)	2.62 × 10^−16^	$1.07\times 10^{5}$

## Data Availability

The data presented in this study are available on request from the corresponding authors.

## References

[1] Monteiro P.J.M., Miller S.A., Horvath A. (2017). Towards sustainable concrete. Nat. Mater..

[2] Mehta P.K., Monteiro P.J.M. (2014). Concrete: Microstructure, Properties, and Materials.

[3] Worrell E., Price L., Martin N., Hendriks C., Meida L.O. (2001). Carbon dioxide emissions from the global cement industry. Annu. Rev. Energy Environ..

[4] Barcelo L., Kline J., Walenta G., Gartner E. (2014). Cement and carbon emissions. Mater. Struct..

[5] Cuesta A., Ayuela A., Aranda M.A.G. (2021). Belite cements and their activation. Cem. Concr. Res..

[6] Salah Uddin K.M., Middendorf B. (2019). Reactivity of Different Crystalline Surfaces of C3S During Early Hydration by the Atomistic Approach. Materials.

[7] Salah Uddin K.M. (2020). Elucidation of Chemical Reaction Pathways in Cementitious Materials.

[8] Odler I. (1991). Strength of cement. Mater. Struct..

[9] Kurdowski W., Duszak S., Trybalska B. (1997). Belite produced by means of low-temperature synthesis. Cem. Concr. Res..

[10] Chi L., Zhang A., Qiu Z., Zhang L., Wang Z., Lu S., Zhao D. (2020). Hydration activity, crystal structural, and electronic properties studies of Ba-doped dicalcium silicate. Nanotechnol. Rev..

[11] Scrivener K.L., John V.M., Gartner E.M. (2018). Eco-efficient cements: Potential economically viable solutions for a low-CO_2_ cement-based materials industry. Cem. Concr. Res..

[12] Izadifar M., Abadi R., Jam A.N., Rabczuk T. (2017). Investigation into the effect of doping of boron and nitrogen atoms in the mechanical properties of single-layer polycrystalline graphene. Comput. Mater. Sci..

[13] Izadifar M., Thissen P., Abadi R., Jam A.N., Gohari S., Burvill C., Rabczuk T. (2019). Fracture toughness of various percentage of doping of boron atoms on the mechanical properties of polycrystalline graphene: A molecular dynamics study. Phys. E Low-Dimens. Syst. Nanostructures.

[14] Salah Uddin K.M., Izadifar M., Ukrainczyk N., Koenders E., Middendorf B. (2022). Dissolution of β-C2S Cement Clinker: Part 1 Molecular Dynamics (MD) Approach for Different Crystal Facets. Materials.

[15] Izadifar M., Ukrainczyk N., Salah Uddin K., Middendorf B., Koenders E. (2022). Dissolution of Portlandite in Pure Water: Part 2 Atomistic Kinetic Monte Carlo (KMC) Approach. Materials.

[16] Salah Uddin K.M., Izadifar M., Ukrainczyk N., Koenders E., Middendorf B. (2022). Dissolution of Portlandite in Pure Water: Part 1 Molecular Dynamics (MD) Approach. Materials.

[17] Izadifar M., Natzeck C., Emmerich K., Weidler P.G., Gohari S., Burvill C., Thissen P. (2022). Unexpected Chemical Activity of a Mineral Surface: The Role of Crystal Water in Tobermorite. J. Phys. Chem. C.

[18] Henkelman G., Uberuaga B.P., Jónsson H. (2000). A climbing image nudged elastic band method for finding saddle points and minimum energy paths. J. Chem. Phys..

[19] Sheppard D., Xiao P., Chemelewski W., Johnson D.D., Henkelman G. (2012). A generalized solid-state nudged elastic band method. J. Chem. Phys..

[20] Henkelman G., Jónsson H. (2000). Improved tangent estimate in the nudged elastic band method for finding minimum energy paths and saddle points. J. Chem. Phys..

[21] Kohn W., Sham L.J. (1965). Self-Consistent Equations Including Exchange and Correlation Effects. Phys. Rev..

[22] Kresse G., Furthmüller J. (1996). Efficient iterative schemes for ab initio total-energy calculations using a plane-wave basis set. Phys. Rev. B.

[23] Izadifar M., Thissen P., Steudel A., Kleeberg R., Kaufhold S., Kaltenbach J., Schuhmann R., Dehn F., Emmerich K. (2020). Comprehensive examination of dehydroxylation of kaolinite, disordered kaolinite, and dickite: Experimental studies and density functional theory. Clays Clay Miner..

[24] Izadifar M., Dolado J.S., Thissen P., Ayuela A. (2021). Interactions between Reduced Graphene Oxide with Monomers of (Calcium) Silicate Hydrates: A First-Principles Study. Nanomaterials.

[25] Kresse G., Furthmüller J. (1996). Efficiency of ab-initio total energy calculations for metals and semiconductors using a plane-wave basis set. Comput. Mater. Sci..

[26] Kresse G., Hafner J. (1993). Ab initio molecular dynamics for liquid metals. Phys. Rev. B.

[27] Izadifar M., Königer F., Gerdes A., Wöll C., Thissen P. (2019). Correlation between Composition and Mechanical Properties of Calcium Silicate Hydrates Identified by Infrared Spectroscopy and Density Functional Theory. J. Phys. Chem. C.

[28] Martin P., Manzano H., Dolado J.S. (2019). Mechanisms and dynamics of mineral dissolution: A new kinetic Monte Carlo model. Adv. Theory Simul..

[29] Martin P., Gaitero J.J., Dolado J.S., Manzano H. (2021). New Kinetic Monte Carlo Model to Study the Dissolution of Quartz. ACS Earth Space Chem..

[30] Lasaga A.C. (2014). Kinetic Theory in the Earth Sciences.

